# Book Reviews

**Published:** 1873-03-15

**Authors:** 


### Book Reviews.

The Practice of Surgery. By Thomas Bryant, F.R.S., Surgeon to Guy's Hospital, London, with 507 illustrations. Published by Henry C. Lea, Philadelphia: 1873.

This octavo volume, of about 1000 pages, is, in many respects, different from the ordinary surgical text-books of English authors. The illustrations, which are over 500 in number, are for the most part original, and copied from specimens in the museum of Guy's Hospital, London. This originality, though not specially advantageous to the undergraduate student, is of great value to the practicing surgeon, as it opens to him a new treasure-house of instructive cases, not found in other books. Another point of the author's superiority over most English writers, is his free use of statistics, which, like his illustrations, are largely from the records of Guy's Hospital. It is unfortunate for science, that British surgeons of eminence early took a prejudice against statistics, and not only neglected them, but even wrote against them. So completely has this feeling prevailed among them that they not unfrequently apologize in a deprecating manner, as though they were committing a sin, when they quote statistical results.

It is probably owing to this influence that, except in Guy's, St. Bartholomew's, St. Thomas's, and the Ophthalmia hospitals, there is scarcely a scrap of useful clinical record to be found among all the numerous hospitals of London. The return of British surgeons to sound sense in this matter will greatly promote the cause of science.

The surgery of the genito-urinary system is quite fully treated, occupying 200 pages. The subject of lithotrity is discussed with especial care.

The author discusses anæsthetics briefly, but with candor and care, so far as chloroform is concerned. He has obtained a more correct idea of the mortality produced by this agent than most other English surgeons. In giving the per cent. of deaths from it he apparently uses the figures of Dr. Andrews, of Chicago, which have recently been republished in the English journals without credit.

He appears to be scarcely acquainted with ether, and merely says, that "the Americans have still a preference" for it.

His discussion of amputation is concise, and marked with excellent sense. At the ankle-joint he prefers Pirogoff's operation to Syme's, contrary to the opinion of most Englishmen, but agreeing with the best surgeons here.

On the whole, this work is to be greatly commended for placing in the hands of the surgeon a mass of new facts, new illustrations, and new opinions, all set forth with candor, and with a clear and a well-balanced judgment.

New books received through Messrs. W. B. Kean & Cook :

A Manual of Histology. By Prof. S. Stricker, of Vienna, Austria. New York: Wm. Wood & Co.

A Treatise on Apoplexy, Cerebral Hæmorrhage, Cerebral Embolism, Cerebral Gout, Cerebral Rheumatism and Epidemic Cerebro Spinal Meningitis. By John A. Lidell, A.M., M.D. New York: Wm. Wood & Co.

Dental Caries, and Its Causes. By Drs. Liebre and Rottenstein. Tsanslated by Thos. H. Chandler, D.M.D. Philadelphia: Lindsay & Blakiston.

